# Limited fibrosis accompanies triple-negative breast cancer metastasis in multiple model systems and is not a preventive target

**DOI:** 10.18632/oncotarget.25231

**Published:** 2018-05-04

**Authors:** Danielle Brooks, Alexandra Zimmer, Lalage Wakefield, L. Tiffany Lyle, Simone Difilippantonio, Fabio C. Tucci, Stephane Illiano, Christina M. Annunziata, Patricia S. Steeg

**Affiliations:** ^1^ Women's Malignancies Branch, Center for Cancer Research, National Cancer Institute, Bethesda, MD, USA; ^2^ Laboratory of Cancer Biology and Genetics, Center for Cancer Research, National Cancer Institute, Bethesda, MD, USA; ^3^ Department of Comparative Pathobiology, Purdue University College of Veterinary Medicine, West Lafayette, IN, USA; ^4^ Laboratory Animal Sciences Program, Leidos Biomedical Research Inc., Frederick National Laboratory for Cancer Research, Frederick, MD, USA; ^5^ Epigen Biosciences, Inc., San Diego, CA, USA; ^6^ Sanofi, Chilly Mazarin, France

**Keywords:** breast cancer, ovarian cancer, fibrosis, lysophosphatidic acid receptor, metastasis

## Abstract

The lysophosphatidic acid receptor 1 (LPAR1) is mechanistically implicated in both tumor metastasis and tissue fibrosis. Previously, metastasis was increased when fulminant fibrosis was first induced in mice, suggesting a direct connection between these processes. The current report examined the extent of metastasis-induced fibrosis in breast cancer model systems, and tested the metastasis preventive efficacy and fibrosis attenuation of antagonists for LPAR1 and Idiopathic Pulmonary Fibrosis (IPF) in breast and ovarian cancer models. Staining analysis demonstrated only focal, low-moderate levels of fibrosis in lungs from eleven metastasis model systems. Two orally available LPAR1 antagonists, SAR100842 and EPGN9878, significantly inhibited breast cancer motility to LPA *in vitro*. Both compounds were negative for metastasis prevention and failed to reduce fibrosis in the experimental MDA-MB-231T and spontaneous murine 4T1 *in vivo* breast cancer metastasis models. SAR100842 demonstrated only occasional reductions in invasive metastases in the SKOV3 and OVCAR5 ovarian cancer experimental metastasis models. Two approved drugs for IPF, nintedanib and pirfenidone, were investigated. Both were ineffective at preventing MDA-MB-231T metastasis, with no attenuation of fibrosis. In summary, metastasis-induced fibrosis is only a minor component of metastasis in untreated progressive breast cancer. LPAR1 antagonists, despite *in vitro* evidence of specificity and efficacy, were ineffective *in vivo* as oral agents, as were approved IPF drugs. The data argue against LPAR1 and fibrosis as monotherapy targets for metastasis prevention in triple-negative breast cancer and ovarian cancer.

## INTRODUCTION

Fibrosis is a chronic wound healing response that has lost normal controls. It is well studied in the lungs (as idiopathic pulmonary fibrosis), the liver, the kidney, and the skin (as systemic scleroderma). After repair of tissue damage, the inflammatory response continues, resulting in the relentless deposition of connective tissue that remodels and destroys normal tissue architecture and function (rev in [1–3]). A key to fibrosis is the permanent activation of myofibroblasts, which express smooth muscle actin (α-SMA) and produce collagen and other extracellular matrix (ECM). Changes in endothelial cells, lymphocytic infiltration and epithelial cells also accompany fibrosis.

The cellular and molecular mechanisms of fibrosis bear an uncanny resemblance to cancer progression (rev in [4]). Shared cell biology characteristics include inflammation, ECM remodeling, proliferation, and the epithelial-mesenchymal transition (EMT). Pathways underlying both processes include TGF-β [5], the receptors to the phospholipid lysophosphatidic acid (LPA) [6], PDGF [7], hedgehog [8], integrins [9], chemokines [4], lysyl oxidase [10], etc. A direct connection was proposed in the transdifferentiation of epithelial cancer cells to fibrosis-inducing myofibroblasts via the EMT [11]. When pulmonary fibrosis was induced in mice by either radiation treatment [12] or bleomycin injection [13, 14], tumor metastasis was significantly accelerated. These data provided the experimental foundation for the hypothesis that fibrosis was a mechanistic contributor to cancer progression and a potential therapeutic target. Human tissue studies have also correlated fibrosis with aspects of metastasis including poor patient survival [15, 16]. Other reports have disagreed, suggesting that fibrosis correlated with better outcome based on changes in immunity [17].

Our previous work centered on the LPA receptor 1 (LPA1). In two breast cancer metastasis models, an LPA1/3 antagonist had no effect on primary tumor growth but significantly prevented the lung and/or liver metastasis of “triple negative” (estrogen and progesterone receptors, ER and PR, negative; HER2 normal) breast cancer. Tumor cells in the distant site treated with the LPA1 antagonist showed reduced proliferation, suggestive of metastatic dormancy [18]. The LPA axis is mechanistically involved in promoting breast cancer metastasis to other sites, and of other cancer types including ovarian cancer [19–21]. The LPA1 antagonist used in our preclinical studies was not orally available, precipitating a search for orally available, biosimilar antagonists that could be used for maintenance cancer therapy in a clinical trial. Several LPA1 antagonists were identified, all coincidentally in development for fibrosis clinical indications, as supported by preclinical literature [6, 22–24].

In the current manuscript, we have further explored the potential connection between fibrosis and cancer metastasis using triple-negative breast cancer and ovarian cancer model systems. We have asked [1] the extent of fibrosis as a result of metastatic colonization in metastatic colonization in model systems; [2] the effect of orally available LPA1 antagonists on metastasis and fibrosis in triple-negative breast cancer model systems; [3] given the association of LPA receptors and ovarian cancer progression [25–27], the effect of these antagonists in ovarian cancer metastasis, and [4] the effect of recently FDA approved drugs for idiopathic pulmonary fibrosis, pirfenidone [28] and nintedanib [29], in a breast cancer model system. The data point to a conclusion that, distinct from experiments where fulminant fibrosis was first induced by chemical agents or radiation, fibrosis is only focally induced in metastasis, and neither orally available LPA1 antagonists nor FDA approved fibrosis drugs provide significant metastasis prevention.

## RESULTS

### Fibrosis in breast cancer metastatic model systems

**Figure 1:**
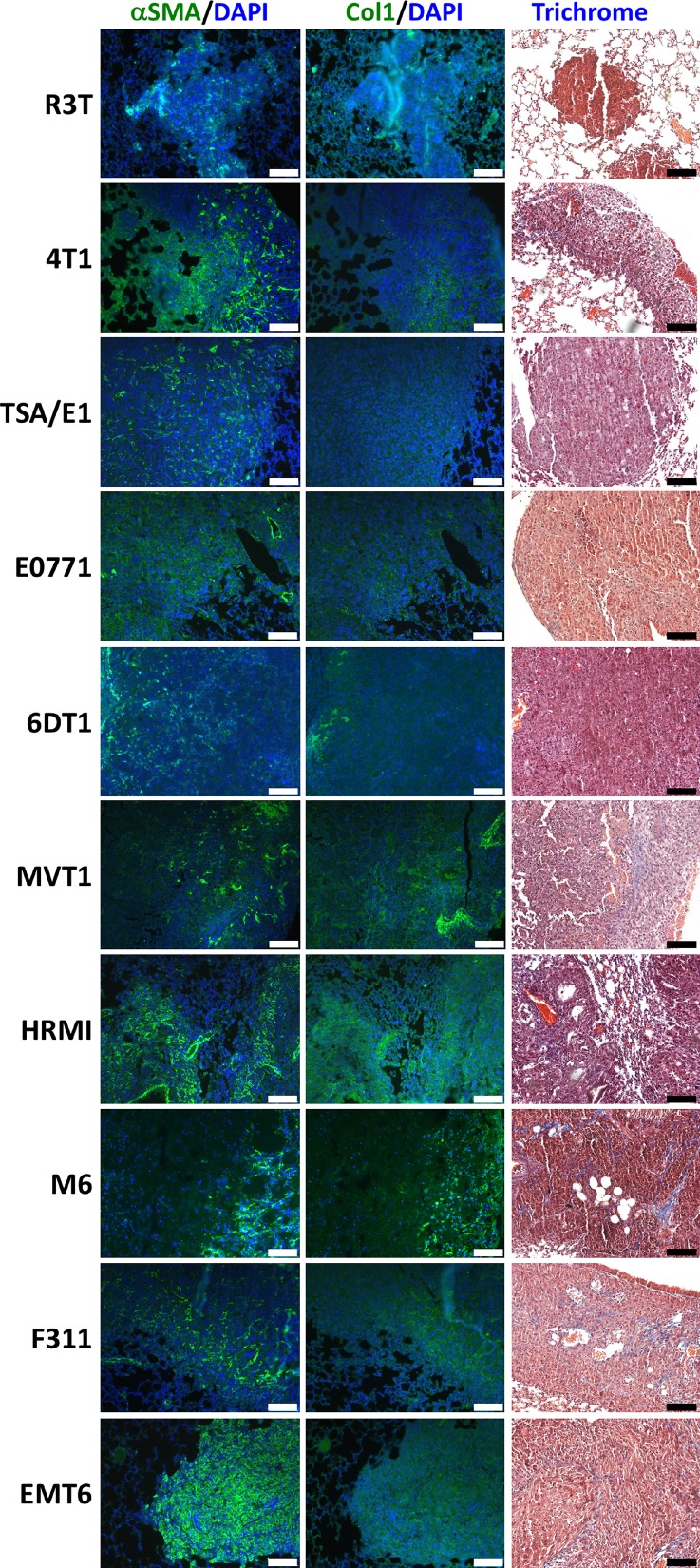
Staining analysis of metastatic lung tissue from ten mouse models of breast cancer Formalin fixed, paraffin embedded (FFPE) lung tissue sections from metastasis bearing mice were stained for presence and extent of fibrosis. Masson's trichrome stain was used to produce an Ashcroft fibrosis score both within the lung metastases (modified Ashcroft score) and the whole lung. Activated fibroblasts were identified by staining for alpha smooth muscle actin (αSMA), and the extracellular matrix was assessed by staining for collagen 1 (Col1). Representative images were obtained at 20× objective and scale bar = 100 μm.

We asked the extent of fibrosis that developed when breast cancer cells metastasize to the lungs of experimental animals. Tissue blocks containing H&E confirmed pulmonary metastatic lesions from ten orthotopic model systems [30] were analyzed for three markers of fibrosis- Masson's trichrome, the stain used in histopathology laboratories, collagen I, and α-SMA. Representative images are shown on Figure 1. Using trichrome stain, the presence of blue tissue indicated fibrosis. Approximately half of the model systems showed evident fibrotic strands dispersed throughout the section. Succeeding tissue sections were stained for collagen 1 and α-SMA. Table 1 presents quantification of the fibrosis staining data by a veterinary pathologist for multiple sections of each model system. IHC staining for molecular subtype and metastatic efficiency data, previously published [30], are listed. We modified the Ashcroft scoring system used for trichrome staining [31], for scoring the models (Supplementary Table 1). On a 0–8 scale, entirely normal tissue was assigned a score of 0, and positive scores were based on the predominate pattern within the microscopic sections. Modified Ashcroft scores were compiled for areas within the metastatic lesions as well as the entire lung (Supplementary Figure 1). In the metastatic lesion, scores in the cohort varied from 0–3 (out of 8). Analysis of the entire lung resulted in scores of 4–5 in half of the model systems. Fibrosis is heterogeneously apparent among the model systems tested, and appears more widespread throughout the lungs than confined to the region of the metastatic lesion. In no model was fibrosis “high” (>6 out of 8). Expression of α-SMA appeared greater than that of the trichrome histochemical stain for fibrosis or collagen I. In general, the models with the highest degree of fibrosis, tended to also have strong α-SMA and collagen I staining, but widespread heterogeneity of markers was observed.

**Table 1:** Summary of immunohistochemical analyses of fibrosis in lung metastases from mouse models of breast cancer^a^

Model:	ER/PR:	HER2:	Metastatic Efficiency:	Modified Ashcroft Score^b^:		α-SMA^c^:	Collagen I^c^:
				Metastasis:	Lung:		
R3T	–/–	Nl	60	0	1	+	–
4T1	–/–	Nl	100	1	3	+++	+
TS/A-E1	+/–	Nl	100	2	2	++	–
E0771	–/–	Nl	50	1	4	+/–	+
6DT1	–/–	Nl	90	1	2	++	++
Mvt1	–/–	Nl	90	1	3	++	+++
HRM1	+/–	Nl	80	1	5	++++	++
M6	–/–	Nl	55	3	5	++++	++++
F3II	–/–	Nl	70	1	5	++	+
EMT6	+/–	Nl	100	1	5	+++++	–/+

^a^Multiple sections of lung metastases were stained for trichrome, α-SMA and collagen I. Model system molecular subtype by immunohistochemistry and metastatic efficiency were previously reported (30).

^b^See Supplementary Table 1. Ranges from 0–8.

^c^Graded on a – to +++++ scale in intensity.

**Figure 2:**
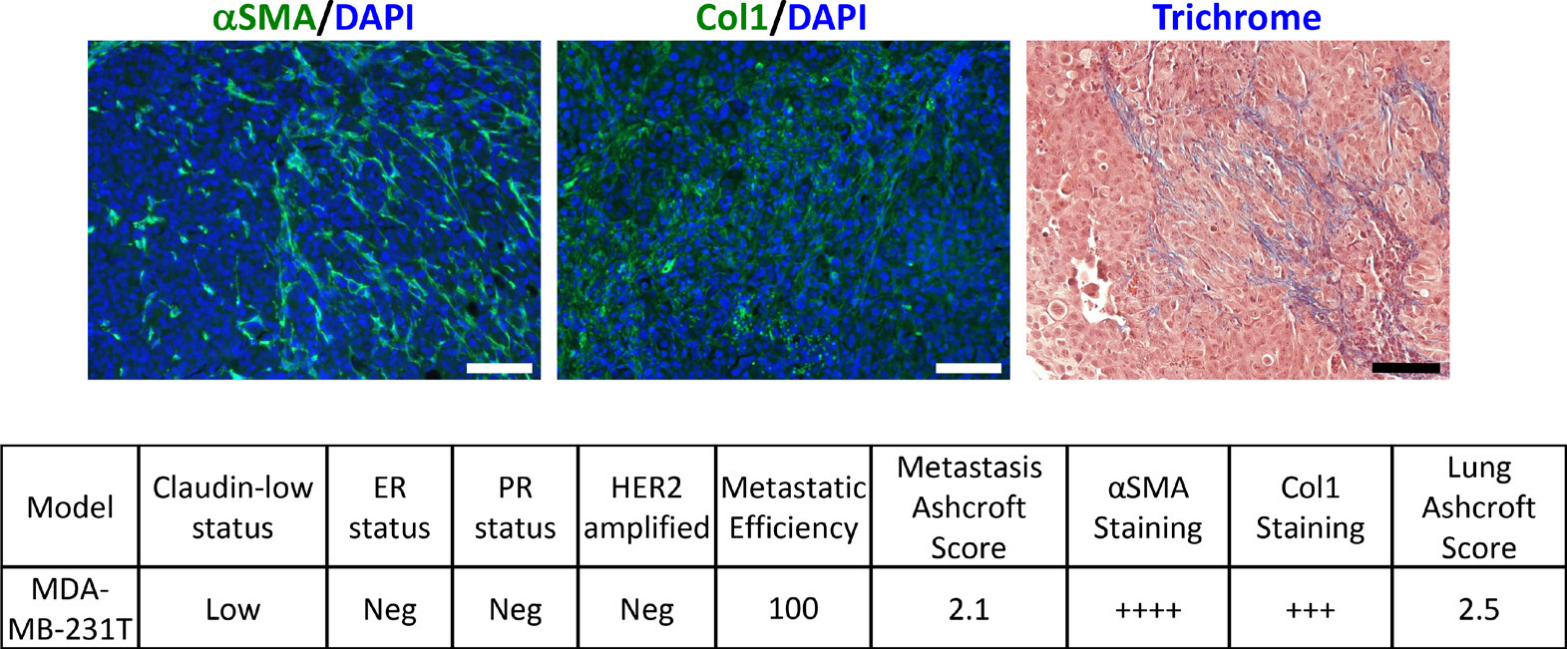
Analysis of fibrosis in metastatic lung tissue from human MDA-MB-231T cells FFPE lung tissue sections were stained for alpha smooth muscle actin (αSMA), Collagen 1 (Col1) or Masson's trichrome to quantify fibrosis in lung metastases from this cell line. Characteristics of the model are listed. Images were taken at 20× objective and scale bar = 100 μm.

Given the widespread use of the MDA-MB-231 triple negative breast cancer model system in translational studies, fibrosis levels were assessed (Figure 2). This human cell line produces lung metastases upon tail vein injection into immunocompromised animals [32]. A relatively low modified Ashcroft score was observed for both the metastatic lesion (2.1) and the entire lung (2.5). Prominent α-SMA staining and moderate collagen I staining were observed.

### LPA1 receptor antagonists as anti-fibrosis agents

We previously reported that a LPA1 antagonist, Debio 0719, significantly prevented metastasis in two models of triple negative breast cancer [18]. LPA1 antagonists are in development both as anti-metastatic agents and fibrosis therapeutics [6, 22–24]. As potential breast cancer therapeutics, LPA1 antagonists would be taken on a maintenance schedule and therefore should be orally available. Also, they should be active in an adjuvant model setting, after primary tumor removal. We tested two orally available LPA1 antagonists: SAR100842 [33] (US Patent 8,362,073 B2) was tested clinically in systemic sclerosis (Clinical Trial Identifier NCT01651143); EPGN9878 is in preclinical development (unpublished).

**Figure 3:**
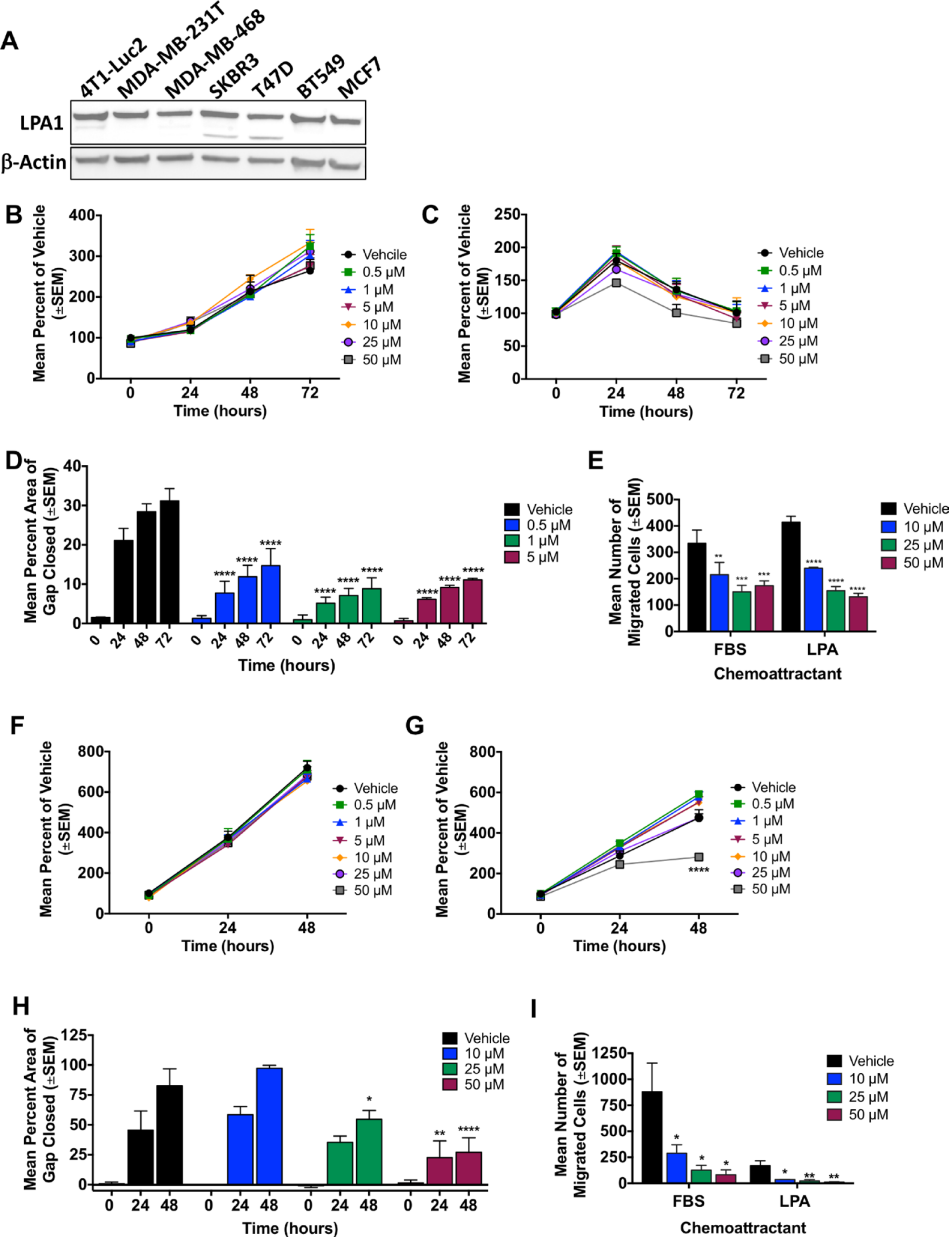
SAR100842 reduced migration and motility of breast cancer cell lines *in vitro* (**A**) Western blot analysis of breast cancer cell lines for LPA1 expression with β-actin as a loading control. (**B**–**C**) Viability of human triple-negative MDA-MB-231T cells with increasing doses of SAR100842 over time in the presence of 10% serum (B) or serum-free media (C). (**D**) MDA-MB-231T cells were measured for the ability to migrate and close a scratch gap in a wound healing assay in the presence of vehicle or increasing concentrations of SAR100842. Percent area of gap that was closed was determined at 24,48 and 72 hours. Two-way ANOVA, *p* < 0.0001 for all concentrations at all time points staring at 24 hours post scratch. (**E**) MDA-MB-231T cells were treated with SAR100842 for 24 hours then plated in a Boyden chamber motility assay and allowed to migrate through a collagen-coated membrane toward either 1% fetal bovine serum (FBS) or 5 μM lysophosphatidic acid (LPA) for 4 hours. ANOVA, *p* = 0.0058, *p* = 0.0003, *p* = 0.0004 for 10 μM, 25 μM and 50 μM respectively to FBS. *P* < 0.0001 for all concentrations to LPA. (**F**–**G**) Viability of murine 4T1-Luc2 mammary carcinoma cells with increasing doses of SAR100842 over time in the presence of 10% serum (F) or serum free media (G). *p* < 0.0001 for 50 μM SAR100842 at 48 hours in serum-free media. (**H**) 4T1-Luc2 cells were measured for the ability to migrate and close a scratch gap in a wound healing assay in the presence of vehicle or increasing concentrations of SAR100842. Two-way ANOVA, *p* = 0.011 for 25 μM at 48 hours, *p* = 0.002 and *p* < 0.0001 for 50 μM at 24 and 48 hours, respectively. (**I**) 4T1-Luc2 cells were treated with SAR100842 for 24 hours then plated in a Boyden chamber motility assay and allowed to migrate through a collagen coated membrane toward either 5% FBS or 10 μM LPA for 4 hours. ANOVA, *p* = 0.04, *p* = 0.02, *p* = 0.01 with 10 μM, 25 μM and 50 μM respectively to FBS and *p* = 0.02, *p* = 0.008, *p* = 0.007 with 10 μM, 25 μM and 50 μM respectively to LPA. All experiments repeated 3-4 times.

LPA1 protein levels were detectable in multiple breast cancer cell lines (Figure 3A). The triple negative breast cancer cell lines MDA-MB-231T and 4T1-Luc2 were used to further test the efficacy of SAR100842 in *in vitro* assays of migration and metastasis. In LPAR1-based *in vitro* inhibition of LPA-stimulated Ca^++^ flux in a cell based assay, SAR100842 had an IC_50_ of 65 nM; and showed no activity up to 10 μM on LPA2, LPA3 or LPA5 in similar calcium assays (data not shown). Increasing doses of SAR100842 did not significantly affect proliferation of either cell line over time (Figure 3B–3C, 3F–3G). However, there was a significant decrease in the ability of cells to migrate in a wound healing assay in a dose dependent manner, 64% reduction (*p* < 0.0001) with 5 μM SAR100842 after 72 hours in MDA-MB-231T and 67% reduction (*p <* 0.0001) with 50 μM SAR100842 after 48 hours in 4T1-Luc2 (Figure 3D and 3H). In a Boyden chamber assay for motility, 50 μM SAR100842 reduced the migration of MDA-MD-231T cells through a collagen membrane by 1.92-fold (*p* = 0.0004) and 3.15-fold (*p <* 0.0001) to FBS and LPA chemoattractants, respectively (Figure 3E). In 4T1-Luc2 cells 50 μM SAR100842 reduced migration by 10.8-fold (*p* = 0.01) and 13.6-fold (*p* = 0.007) to FBS and LPA, respectively (Figure 3I). The data demonstrate an inhibition of a metastasis and fibrosis associated target at μM concentrations *in vitro*.

**Figure 4:**
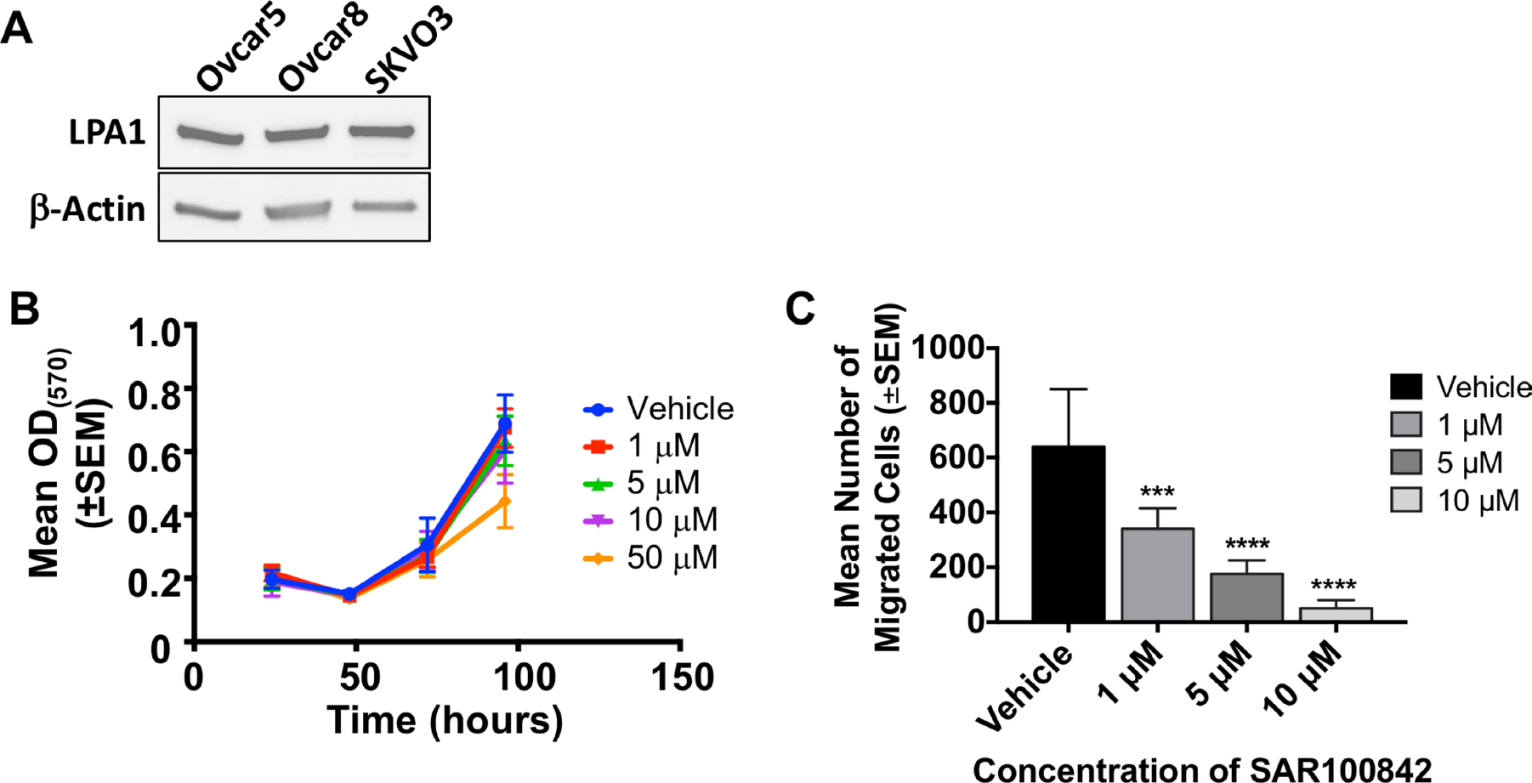
SAR100842 reduced motility of ovarian cancer cell lines *in vitro* (**A**) Western blot analysis of ovarian cancer cell lines for LPAR1 expression with β-actin as a loading control. (**B**–**C**), Viability and motility of SKOV3 cells as described in legend to Figure 3. (B) Viability in 10% serum. (C) Motility toward 5 μM LPA for 4 hours. ANOVA, *p* = 0.0006 for 1 μM, *p* < 0.0001 for 5 and 10 μM. All experiments repeated in triplicate.

As LPA1 is also associated with ovarian cancer progression [27, 34, 35], three ovarian cancer cell lines were analyzed for LPA1 expression as well (Figure 4A). The SKVO3 line was used for further *in vitro* analysis. There was no effect with SAR100842 on proliferation over time when compared to vehicle (Figure 4B). There was a 12.8-fold (*p* < 0.0001) reduction in motility with 10 μM SAR100842 with LPA as a chemoattractant (Figure 4C). Testing of SAR100842 then proceeded to animal models using the same lines.

**Figure 5:**
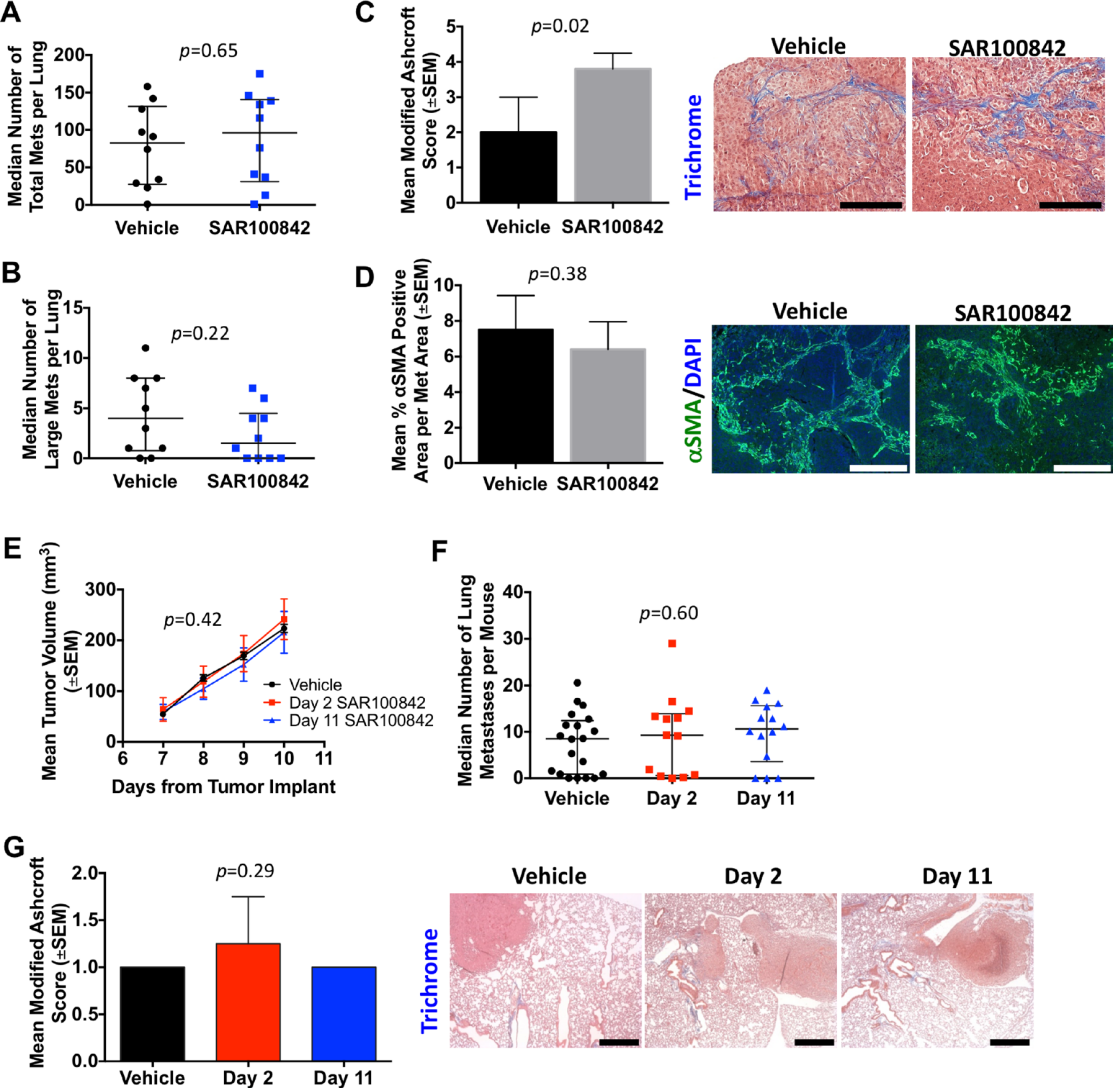
SAR100842 did not prevent metastases or attenuate fibrosis in two mouse models of metastatic triple-negative breast cancer (**A**) Lungs bearing metastases from MDA-MB-231T cells injected in the tail vein were fixed in Bouin's solution. Surface metastases for vehicle and 30 mg/kg SAR100842 treated mice, median ± interquartile range, Mann-Whitney *t*-test, *p* = 0.65. (**B**) Large surface metastases were considered any metastasis lesion that measured greater than 5 mm, median ± interquartile range, Mann-Whitney *t*-test, *p* = 0.22. (**C**) FFPE lungs were sectioned and stained with Masson's trichrome. The mean modified Ashcroft score from each mouse lung is plotted. *N* = 5 mice per group. Images were taken at 20× objective and scale bar = 200 μm. Mann-Whitney *p* = 0.22. (**D**) Lung sections were stained with αSMA. The αSMA positive area of metastases over total metastases area in field was tabulated. *N* = 5 mice per group. Images were taken at 10×, scale bar = 400 μm. Unpaired *t*-test, *p* = 0.38. (**E–F**) 4T1-Luc2 cells were implanted into the #4 mammary fat pad. Mice were randomized into three groups and treatment of vehicle or 30 mg/kg SAR100842 started on day 2 for groups one and two to continue through to endpoint. Tumors were palpated daily and measurements started on day 7. All groups had tumors removed in a survival surgery on day 10. (**E**) Primary tumor size Primary tumor size, Two-way ANOVA, *p* = 0.42. On day 11, one day after primary tumor removal, group 3 began treatment with 30 mg/kg SAR100842. At endpoint of day 70, all lungs were collected. (**F**) The median number of lung metastases per mouse is plotted (± interquartile range). *N* = 15–20 mice per group. ANOVA, *p* = 0.60. (**G**) One section representing the largest lung surface area was stained with Masson's trichrome. Fibrosis was scored in 5 fields. The mean modified Ashcroft score per mouse is plotted. *N* = 15–20 mice per group. Kruskal-Wallis, *p* = 0.29. Images were taken at 5× objective, scale bar = 500 μm.

In the MDA-MB-231T experimental metastasis model system, treatment with SAR100842 showed no difference in total metastasis count (Figure 5A, medians of 83 and 96 in controls and SAR100842, respectively, *p* = 0.65). The median number of large pulmonary metastases (>5 mm) was lower in SAR100842 treated mice (medians of 4 and 1.5 in controls and SAR100842, respectively) but statistically insignificant (*p* = 0.22). Masson's trichrome staining of the lungs indicated an increase in fibrosis in SAR100842 treated mice when analyzed by modified Ashcroft scoring (*p* = 0.02). However, no significant change in α-SMA staining was observed.

When SAR100842 was dosed in animals bearing 4T1 tumor cells, there was no effect on primary tumor size (Figure 5E, -8.0% inhibition in day 2 versus vehicle and 3.5% inhibition in day 11 versus vehicle, *p* = 0.42). The number of lung metastases, determined by histological analysis of H&E stained step sections, was unchanged (Figure 5F, medians of 8.5, 9.3, and 10.6 in the vehicle, day 2 and day 11 treatment groups respectively, *p* = 0.60). Consistent with the relatively low background levels of fibrosis in the 4T1 model (Table 1), trichrome staining of the lungs from this experiment revealed overall low modified Ashcroft scores that did not vary with treatment (Figure 5G).

The effect of SAR100842 on ovarian cancer metastasis was explored in two model systems, SKOV3 and OVCAR5. The outgrowth of metastatic deposits on tissues, either as surface tumors or invasive lesions, was the primary endpoint (Table 2). In the vehicle control groups, metastatic deposits were common on the surface of multiple organs including the omentum, liver, diaphragm, and peritoneum; invasive lesions were only common in the diaphragm. Ascites were rare. Only a weak reduction in diaphragm, kidney and lymph node metastases was evident in the SKOV3 model treated early with SAR100842; none of these reductions were maintained in the group that started treatment on day 10 post-injection. For the OVCAR5 model, metastases were observed at day 70 post-injection on the omentum, liver, diaphragm, pancreas and peritoneum. Of these, SAR100842 apparently reduced diaphragm tumor deposits, both surface and invasive, but was without significant effect in other locations. Ascites formed with this model, but the numbers were too low for any trend to be apparent.

**Table 2:** Effect of SAR100842 on ovarian cancer metastasis incidence in two model systems^a^

Site:	Lesion Depth, where applicable:	Fraction of mice with metastases^b^:					
		SKOV3 Model System:			OVCAR5 Model System:		
		Vehicle:	SAR100842: Day 2 pi	SAR100842: Day 10 pi	Vehicle:	SAR100842: Day 2 pi	SAR100842: Day 10 pi
Omentum		13/13	10/10	10/10	9/12	8/10	7/10
Liver	Surface	11/13	9/10	9/10	9/12	9/10	6/10
	Invasive	1/13	0/10	0/10	2/12	2/10	1/10
Diaphragm	Surface	12/13	7/10	10/10	7/12	5/10	4/10
	Invasive	12/13	7/10	10/10	7/12	5/10	2/10
Lymph Node	Surface	4/13	2/10	3/10	0/12	1/10	3/10
	Invasive	0/13	0/10	0/10	0/12	0/10	0/10
Kidney	Surface	5/13	1/10	2/10	0/12	0/10	0/10
	Invasive	0/13	0/10	0/10	0/12	0/10	0/10
Pancreas	Surface	0/13	0/10	0/10	7/12	8/10	6/10
	Invasive	0/13	0/10	0/10	5/12	5/10	4/10
Peritoneal	Surface	13/13	10/10	10/10	11/12	9/10	7/10
	Invasive	0/13	0/10	0/10	0/12	0/10	0/10
Ascites		0/13	0/10	0/10	3/12	1/10	1/10

^a^Mice were injected intraperitoneally with either SKVO3 or OVCAR5 and treated with either vehicle or 30 mg/kg SAR100842 twice daily starting on either day 2 or day 10 post cell injection (pi). At endpoint, all organs thought to harbor tumor were fixed in 10% NBF before embedding in paraffin for sectioning and H&E staining.

^b^Metastases were quantified in three sections every 200 μm through each tissue, and characterized as being on the surface of the tissue or invading into the tissue, in consultation with a pathologist.

A second potential LPA1 antagonist is EPGN9878 (US 2016/0024031A1). This orally available LPAR1 antagonist is currently in development for renal fibrosis. In LPAR1-based *in vitro* inhibition of LPA-stimulated Ca^++^ flux in a cell based assay, EPGN9878 had an IC_50_ of 8 nM; an LPA3 assay showed an IC_50_ of >10,000 nM. *In vivo* EPGN has a half-life of 3.5 h and a C_max_ of 859 ng/mL after a 20 mg/kg oral dosing in mice (personal communication, Dr. Fabio Tucci, Epigen Biosciences).

**Figure 6:**
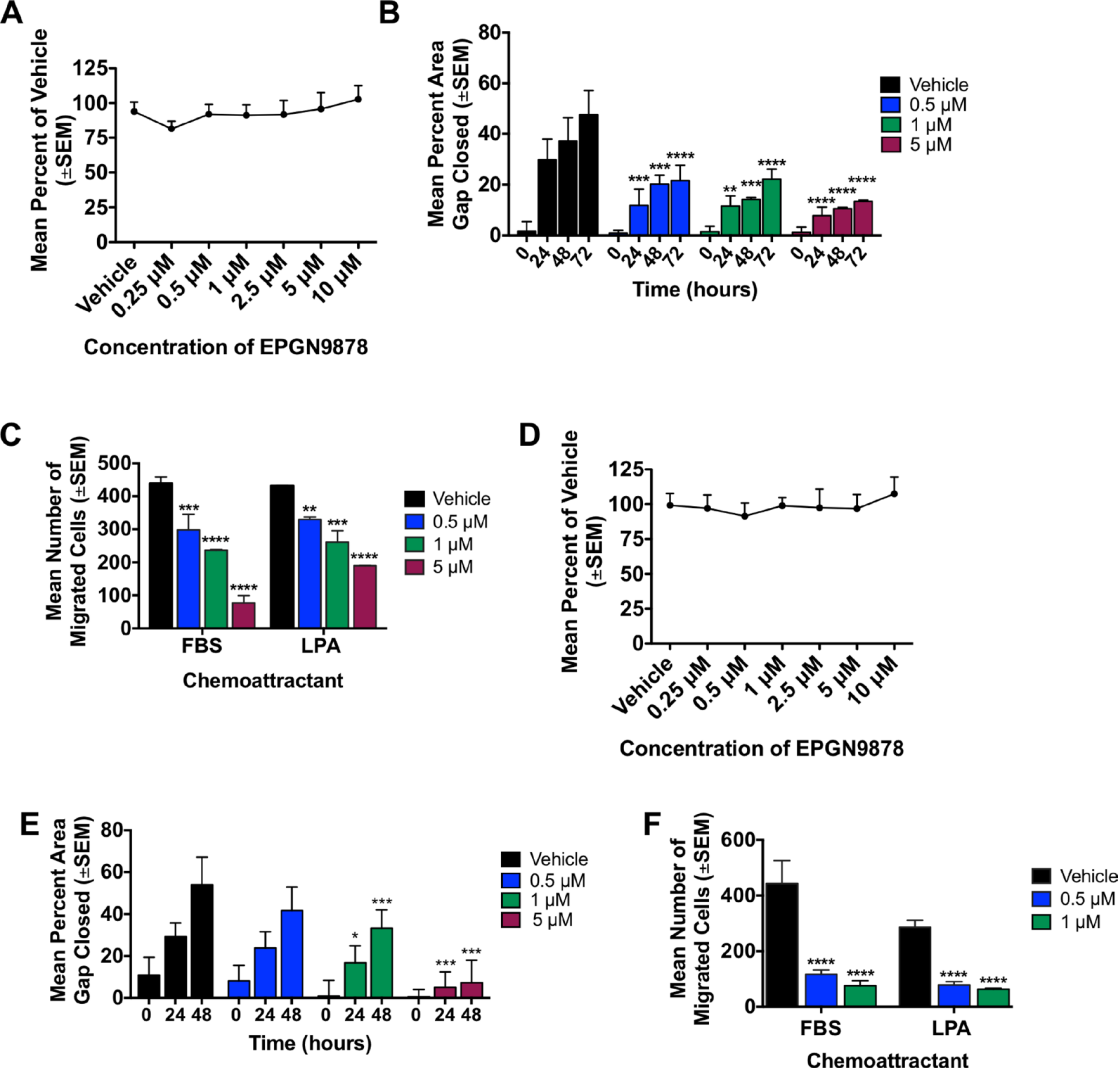
LPA1 antagonist, EPGN9878, reduced migration and motility of triple-negative breast cancer cell lines Viability, motility and migration analyses were performed as described in legend to Figure 3. (**A**) Viability of MDA-MB-231T cells with increasing doses of EPGN9878 after 72 hours in the presence of 10% serum. (**B**) Scratch wound closure of MDA-MB-231T cells in the presence of vehicle or increasing concentrations of EPGN9878. Two-way ANOVA, *p* < 0.005 for all concentrations at 24 hours, *p* < 0.0003 at all concentrations for 48 hours, and *p* < 0.0001 at all concentrations at 72 hours. (**C**) MDA-MB-231T cells were treated with EPGN9878 for 24 hours then plated in a Boyden chamber motility assay and allowed to migrate through a collagen-coated membrane toward either 1% FBS or 5 μM LPA for 4 hours. ANOVA, *p* = 0.0008, *p* < 0.0001, *p* < 0.0001 for 0.5 μM, 1 μM and 5 μM respectively to FBS. *P* = 0.006, *p* = 0.0002, *p* < 0.0001 for 0.5 μM, 1 μM and 5 μM respectively to LPA. (**D**) Viability of 4T1-Luc2 cells with increasing doses of EPGN9878 after 72 hours in the presence of 10% serum. (**E**) 4T1-Luc2 scratch assay closure in the presence of vehicle or increasing concentrations of EPGN9878. Two-way ANOVA, *p* < 0.05 with 1 μM at 24 and 48 hours, *p* < 0.003 with 5 μM at 24 and 48 hours. (**F**) 4T1-Luc2 cells were treated with EPGN9878 for 24 hours then plated in a Boyden chamber motility assay and allowed to migrate through a collagen-coated membrane toward either 5% FBS or 10 μM LPA for 4 hours. ANOVA, *p* < 0.0001 for 1 and 5 μM to both FBS or LPA. All experiments repeated 2–3 times.

EPGN9878 was first tested in MDA-MB-231T and 4T1-Luc2 cell lines. There was no change in cell viability for either cell line with increasing concentrations of EPGN9878 after 72 hours compared to vehicle (Figure 6A and 6D). In a wound healing assay 5 μM of EPGN9878 decreased the migration of cells to close the gap by 72% (*p* < 0.0001) and 87% (*p* < 0.003) in MDA-MB-231T cells at 72 hours and 4T1-Luc2 cells at 48 hours, respectively (Figure 6B and 6E). In MDA-MB-231T cells motility in a Boyden chamber assay was reduced by 5.7-fold to FBS and 2.3-fold to LPA chemoattractants with 5 μM EPGN9878 (Figure 6C, *p* < 0.0001). Similar results were seen in 4T1-Luc2 cells with a reduction in motility of 5.7-fold and 4.5-fold (Figure 6F, *p* < 0.0001). These data provided evidence of LPA signaling inhibition in motility *in vitro*.

**Figure 7:**
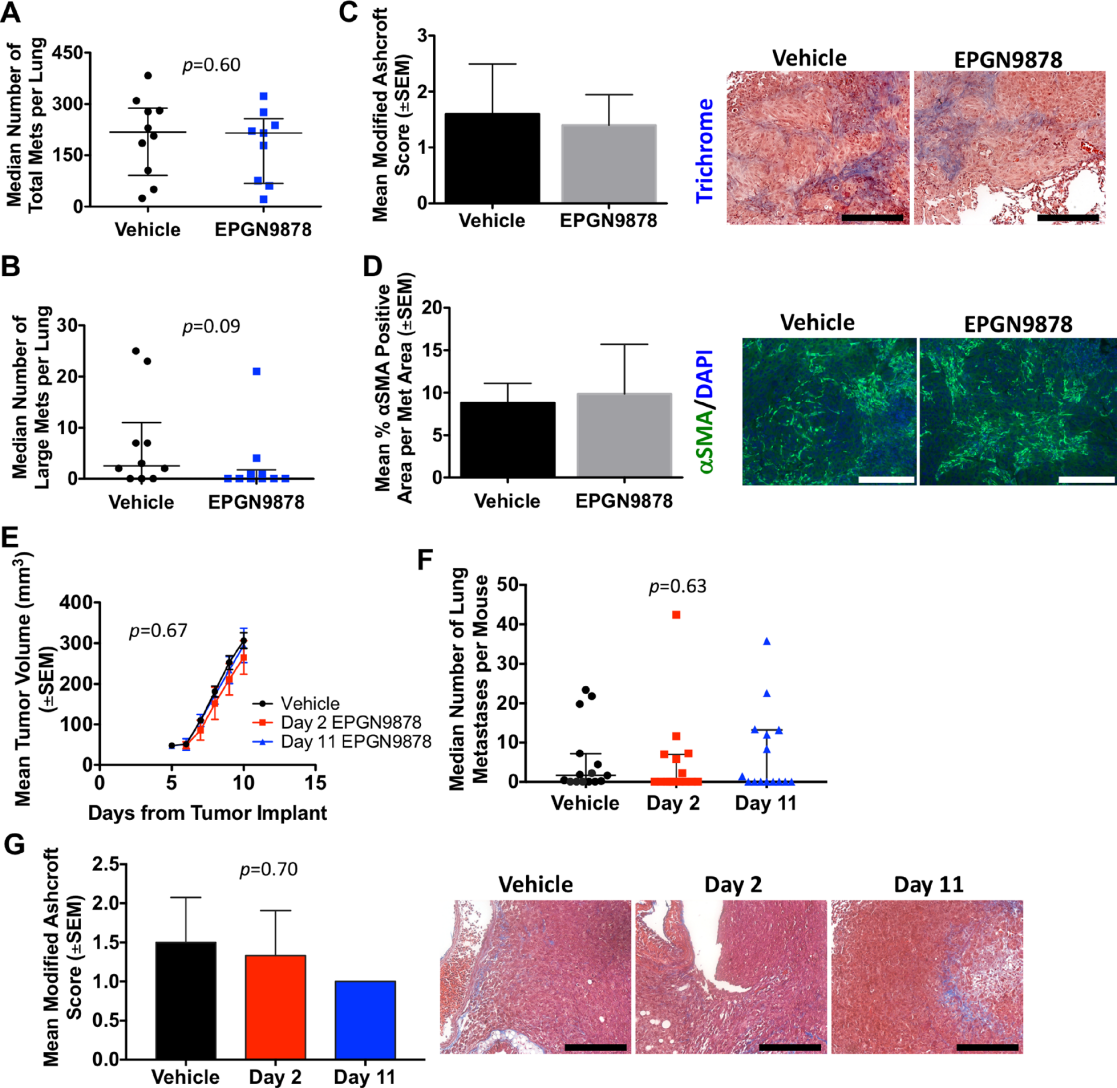
EPGN9878 did not prevent metastases or attenuate fibrosis in two mouse models of metastatic triple-negative breast cancer Metastasis assays and endpoints were described in legend to Figure 5. (**A**) MDA-MB-231T surface metastases for vehicle and 40 mg/kg EPGN9878 treated mice. Median ± interquartile range, Mann-Whitney *t*-test, *p* = 0.60. (**B**) Large surface metastases, median ± interquartile range, Mann-Whitney *t*-test, *p* = 0.09. (**C**) FFPE lungs sections stained with Masson's trichrome. The mean Ashcroft score from each mouse lung is plotted. *N* = 5 mice per group. Images were taken at 20× objective and scale bar = 200 μm. Mann-Whitney *p* = 0.99. (**D**) Lung sections were stained with αSMA. The mean percent positive αSMA per metastasis area, *N* = 5 mice per group. Images were taken at 20×, scale bar = 200 μm. Unpaired *t*-test, *p* = 0.75. (**E**) 4T1-Luc2 spontaneous metastasis assay comparing vehicle or 40 mg/kg EPGN9878 started on day 2 or 11. Primary tumor size, Two-way ANOVA, *p* = 0.67. (**F**) The median number of lung metastases per mouse from day 70 is plotted (± interquartile range). *N* = 15–20 mice per group. ANOVA, *p* = 0.63. (**G**) One section representing the largest lung surface area was stained with Masson's trichrome. The mean modified Ashcroft score per mouse is plotted. *N* = 15–20 mice per group. Kruskal-Wallis, *p* = 0.70. Images were taken at 20× objective, scale bar = 200 μm.

In the MDA-MB-231 experimental metastasis model, total surface lung metastases were comparable between control and EPGN9878 treated groups (Figure 7A, medians of 218 and 215, *p* = 0.60). A trend toward reduced large metastases (>5 mm) was observed, with a median of 2.5 large metastases in the control arm and 0 in the EPGN9878 arm (Figure 7B, *p* = 0.09). No differences in lung fibrosis were observed using Masson's trichrome or α-SMA staining (Figure 7C–7D).

In the 4T1 model, EPGN9878 treatment beginning on either day 2 or day 11, had no effect on primary tumor size or pulmonary metastases detected in step sections (Figure 7E–7F). Staining for fibrosis of the lungs was comparable in all experimental arms (Figure 7G).

In summary, two orally available LPA1 antagonists showed *in vitro* activity consistent with an anti-metastatic effect, such as motility inhibition. However, *in vivo* metastasis preventive activity in either triple-negative breast or ovarian cancers was insufficient to justify additional translation efforts toward clinical testing. Consistent with the metastasis data, the two LPA1 antagonists had no discernable effect on tissue fibrosis.

### FDA approved drugs for idiopathic pulmonary fibrosis

**Figure 8:**
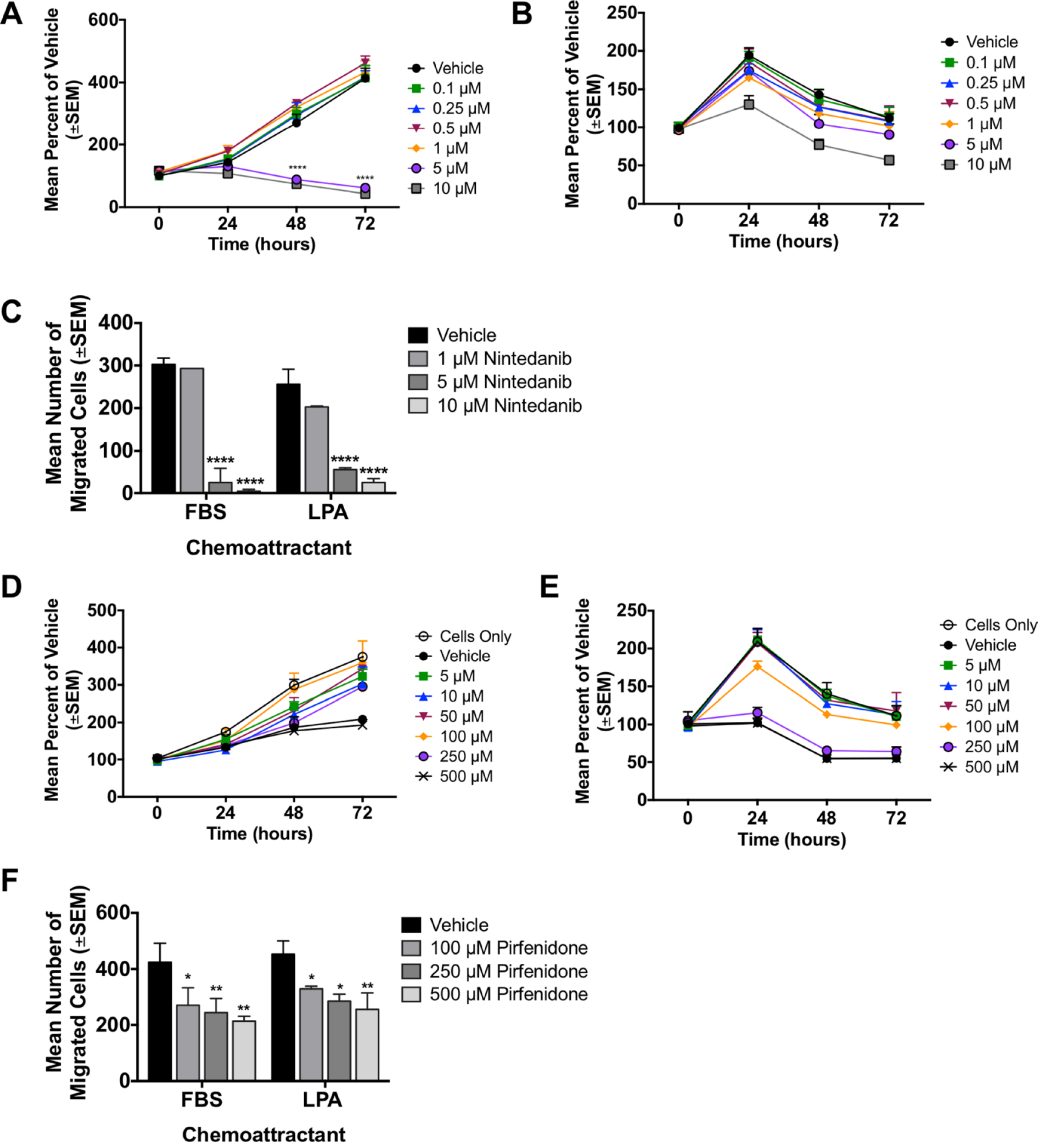
*In vitro* characterization of two idiopathic pulmonary fibrosis (IPF) drugs (**A**–**B**) Viability of MDA-MB-231T cells with increasing doses of nintedanib over time in the presence of 10% serum (A) or serum free media (B). Two-way ANOVA, *p* < 0.0001 with 5 and 10 μM nintedanib at 48 and 72 hours. (**C**) MDA-MB-231T cells were treated with nintedanib for 24 hours then plated in a Boyden chamber motility assay and allowed to migrate through a collagen coated membrane toward either 1% FBS or 5 μM LPA for 4 hours. ANOVA, *p* < 0.0001 for 5 μM and 10 μM to both FBS and LPA. (**D**–**E**) Viability of MDA-MB-231T cells with increasing doses of pirfenidone over time in the presence of 10% serum (D) or serum free media (E). (**F**) MDA-MB-231T cells were treated with pirfenidone for 24 hours then plated in a Boyden chamber motility assay and allowed to migrate through a collagen coated membrane toward either 1% FBS or 5 μM LPA for 4 hours. ANOVA, *p* = 0.019, *p* = 0.003, *p* = 0.002 for 100 μM, 250 μM and 500 μM, respectively to FBS. *P* = 0.036, *p* = 0.011, and *p* = 0.003 for 100 μM, 250 μM and 500 μM, respectively to LPA.

**Figure 9:**
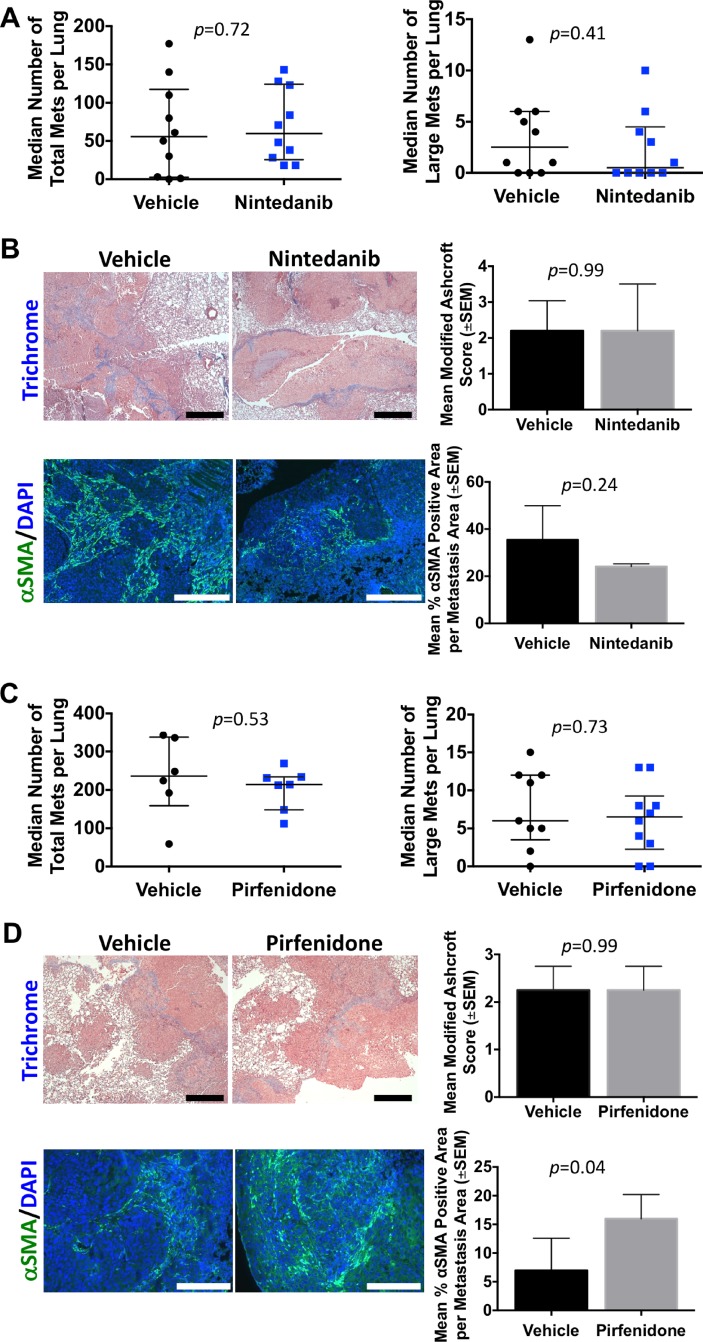
Lack of breast cancer metastasis prevention or attenuation of fibrosis in two drugs approved for idiopathic pulmonary fibrosis Metastasis assays conducted as described in the legend to Figure 5. (**A**) MDA-MB-231T surface metastases for vehicle and 50 mg/kg nintedanib treated mice, median ± interquartile range, Mann-Whitney *t*-test, *p* = 0.72. Large surface metastases, median ± interquartile range, Mann-Whitney *t*-test, *p* = 0.41. (**B**) Modified Ashcroft score of FFPE lung sections, *N* = 5 mice per group. Images were taken at 5× objective and scale bar = 500 mm. Mann-Whitney *p* = 0.99. Serial lung sections stained with aSMA, *N* = 5 mice per group. Images were taken at 10×, scale bar = 400 mm. Unpaired *t*-test, *p* = 0.24. (**C**) Total surface MDA-MB-231T metastases were counted for vehicle and 250 mg/kg pirfenidone treated mice. Median ± interquartile range, Mann-Whitney *t*-test, *p* = 0.53. Large surface metastases, median ± interquartile range, Mann-Whitney *t*-test, *p* = 0.73. (**D**) Lungs were analyzed for fibrosis using the modified Ashcroft score on trichrome stained lung sections, *N* = 5 mice per group. Images were taken at 20× objective and scale bar = 200 mm. Mann-Whitney *p* = 0.99. Serial lung sections were stained with aSMA. The mean percent positive aSMA per met area is plotted. *N* = 5 mice per group. Images were taken at 20×, scale bar = 200 mm. Unpaired *t*-test, *p* = 0.04.

During the conduct of our LPA/fibrosis studies, two unrelated drugs were approved for the treatment of lung fibrosis, using respiratory capacity as the primary clinical endpoint. Nintedanib is a multi-tyrosine kinase inhibitor. In randomized trials, nintedanib significantly slowed patient declines in forced vital capacity [29]. *In vitro*, nintedanib significantly reduced the proliferation of MDA-MB-231T cells at concentrations of 5 and 10 μM at 48 and 72 hours (Figure 8A–8B, *p* < 0.0001). Additionally, motility was significantly reduced (*p* < 0.0001) at the same concentrations (Figure 8C). We determined the effect of nintedanib on lung metastases in the MDA-MB-231 experimental metastasis model (Figure 9A–9B). Surface pulmonary metastases were comparable between the vehicle and nintedanib arms. A minor reduction in large metastases (>5 mm) was observed, from a median of 2.5 in the control arm to 0.5 in the nintedanib arm, without statistical significance (*P* = 0.41). Analysis of lung fibrosis showed no change in modified Ashcroft score, but a trend of decreased α-SMA staining in the nintedanib treated arm (median of 35.5 in the vehicle versus 24.1 in the nintedanib arm, *P* = 0.24).

A second approved drug for idiopathic pulmonary fibrosis is pirfenidone, also with a primary endpoint of forced vital capacity in idiopathic pulmonary fibrosis [28]. Compared to vehicle control pirfenidone did not significantly decrease the proliferation of MDA-MB-231T cells. However, the vehicle did have a slight cytotoxic effect on the cells themselves when compared to untreated cells (Figure 8D–8E). Pirfenidone significantly reduced motility in a dose-dependent manner to both FBS and LPA chemoattractants (Figure 8F). *In vivo*, comparable numbers of pulmonary metastases were observed between vehicle and pirfenidone treated mice. A minor change in large metastases (>5 mm) was observed, from a median of 6 in the vehicle arm to a median of 6.5 in the pirfenidone arm, statistically insignificant (*P* = 0.73). Staining of the lung tissues for fibrosis showed no difference in the arms using trichrome stain, but a trend of increased α-SMA staining in the pirfenidone arm (*P* = 0.04).

In conclusion, neither of two FDA approved drugs for idiopathic pulmonary fibrosis was metastasis preventive in the MDA-MB-231 triple-negative model system, nor was fibrosis attenuated.

## DISCUSSION

While fibrosis is a clear stimulant of metastasis in mouse models in which fulminant conditions are first induced, its relevance to metastasis under normal physiological conditions is less clear. We find, among multiple breast cancer model systems, focal and heterogeneously low levels of fibrosis occur in lung metastases, with heterogeneous, moderate levels in the general lung. How these data compare to breast cancer patient tissues is difficult to assess due to an inadequate literature, largely focused on primary tumors. In the largest study, among 1850 patients undergoing two types of adjuvant therapy, central necrosis and fibrosis was only found in 3.6–4.9% of patients’ primary tumors, but was strongly correlated with a triple-negative histology; central necrosis and fibrosis correlated with poor outcome [36]. Analysis of α-SMA expression by myofibroblasts showed heterogeneity among 60 primary breast tumors, with a mean of 8.5% of tumor area positive; higher fibrosis levels correlated with poor outcome [37] . Data for untreated metastatic sites is lacking. Radiation therapy at the primary site and lymphatics is associated with local skin fibrosis and occasional reports exist for fibrosis in the underlying lungs [38, 39]. Indications of fibrosis have been described in peritoneal masses from ovarian cancer but little quantification has been reported [40, 41]. Further data from metastatic biopsies or autopsies would be useful. It remains possible that other cancer types may have higher levels of fibrosis in metastases, such as pancreatic cancer [42] and would constitute a better therapeutic window for anti-fibrosis agents. It also remains possible that other measures of fibrosis may show more pronounced alterations.

The LPA pathway has been demonstrated to induce fibrosis [43] and antagonists have also been credentialed [6, 44]. Our laboratories and others reported that the LPA pathway stimulates breast [21, 45, 46] and ovarian [25, 34, 47] cancer metastasis, and LPAR1 antagonists significantly prevented lung, liver and bone metastasis formation [18, 48]. Metastasis formation has been ascribed to both tumor intrinsic and microenvironmental effects of the LPA pathway [49]. At the intersection of these two phenotypes, fibrosis has been reported as a “soil” for the seeding of metastases [50, 51], expansion of micrometastases [16], promotion of tumor viability [10], and immune effects [17], suggesting the hypothesis that LPA-induced fibrosis may be a mechanism of metastasis promotion. This hypothesis was contrary to published data where breast cancer cells with a LPA1 knockdown were metastasis suppressed [18], suggesting a tumor cell-intrinsic effect, but did not preclude an indirect microenvironmental effect or a transdifferentiation of carcinoma cells to myoepithelial cells [11]. Testing of this hypothesis was important to both understand the biology of metastasis and explore translational avenues. Testing of LPA1 antagonists via oral gavage was considered critically important, as any clinical antagonist would need to be given on a continuous basis. Also, a clinical trial would likely enroll patients at high risk for metastasis, but where metastatic foci were not observable, the adjuvant setting. We modeled the adjuvant setting by not beginning drug administration immediately after initial tumor cell injection, when micrometastases were likely formed. Two orally available LPA1 antagonists (SAR100842, EPGN9878) were without significant effects on metastasis suppression or extent of fibrosis in two triple-negative breast cancer model systems. Hints of activity were observed with the reduction of large metastases using either SAR100842 or EPGN9878 in the MDA-MB-231 model system, but were not robust and failed to repeat in adjuvant setting models, where compounds were administered after micrometastases have developed.

The LPA pathway is extensively credentialed in ovarian cancer initiation and progression [25, 34, 52–55]. In gastric cancer, a connection was made between peritoneal fibrosis and peritoneal metastasis [50]. Ovarian preclinical metastasis prevention experiments were therefore conducted with SAR100842. In these experiments two cell lines were used, and produced surface and invasive lesions in multiple peritoneal organs, in addition to ascites. The quantification of invasive lesions was considered important, as these are the lesions that are measured for clinical responses in trials. While the LPA pathway is extensively credentialed in ovarian cancer initiation and progression, SAR100842 was ineffective in preventing metastasis in two models suggesting that contribution of other LPARs is important in these cells beyond LPA1.

Multiple factors may contribute to these preclinical failures. The LPA1 receptor was expressed by all of the cell lines used. Both compounds demonstrated a significant reduction in LPA stimulated tumor cell motility, suggesting that each compound could hit the intended target *in vitro*. However, further optimization of a dosing regimen based on pharmacokinetic assessment and modeling would be needed to ensure that the receptor target is blocked at all times during the study. Another orally active LPA1 antagonist was reported with preclinical activity in pancreatic cancer (which have been shown to have higher levels of fibrosis), suggesting low levels of fibrosis in breast and ovarian cancers may be responsible herein [56]. It is also known that fibrosis is mediated by a host of molecular pathways; LPA1 may only be one of many contributors. TGF-β [57, 58] and other pathways [8, 59] are under evaluation for fibrosis. Finally, the definition of fibrosis varies between reports. We used the trichrome clinical assay, but other labs report that collagen crosslinking [10], TGF-β signaling, and other aspects of fibrosis may be better therapeutic targets.

To ask the question of the relationship of fibrosis and metastasis more broadly, we investigated two drugs recently FDA approved for idiopathic pulmonary fibrosis. Nintedanib is a multi-tyrosine kinase inhibitor targeting FGFR, PDGFR and VEGFR. It was credentialed in preclinical models of lung fibrosis and systemic sclerosis and slowed the decline of forced vital capacity in idiopathic pulmonary fibrosis patients [29, 60, 61]. Pirfenidone has both anti-fibrosis and anti-inflammatory properties through incompletely defined mechanisms that may include the TGF-β pathway and proteases. It also inhibited fibrosis preclinically and slowed the decline of forced vital capacity in patients [28, 62, 63]. Using the MDA-MB-231 triple negative breast cancer model, neither drug had significant effects on metastasis formation or extent of fibrosis. Takai *et al*. also studied pirfenidone in the 4T1 mouse model of triple negative breast cancer. *In vitro*, pirfenidone inhibited the viability and collagen production of carcinoma activated fibroblasts. *In vivo*, pirfenidone was without activity on tumor growth or metastasis as monotherapy, similar to our data in the MDA-MB-231 model, however an inhibition of fibrosis assayed by pico-sirius red staining was observed [64]. For both drugs, their multi-faceted mechanisms of action suggest that non-fibrosis effects may also occur, for instance inhibition of tumor cell receptor tyrosine kinase signaling. In conclusion, general anti-fibrosis drugs show little anti-metastatic activity in preclinical models and inconsistent effects on fibrosis levels.

Fibrosis may be important in other parts of breast and ovarian cancer progression such as primary tumor formation or the shedding of tumor cells. This scenario is not modeled by our preclinical experiments and would be more germane to cancer prevention trials. In both breast and ovarian cancers it is thought that tumor cell shedding can occur before patient diagnosis and surgery and is therefore not an optimal clinical trial target.

Our data suggest that, while fibrosis occurs in metastatic sites in animal models of breast and ovarian cancers, it is either insufficient or inadequately drugged to be a metastasis preventive target. The strengths of this work lie in the multiple model systems and preclinical compounds used, a reliance on the clinical assay for fibrosis, and experimental designs that attempted to mimic clinical trials. A limitation of the work is a lack of inclusion of drug and radiation therapy in the model systems. Increased fibrosis has been reported after chemotherapy with fluoropyrimidines [65], methotrexate [66] and paclitaxel [67], and radiation therapy. If greater amounts of fibrosis occur *in vivo* at metastatic sites, this may indicate promise for the heavily pretreated, metastatic clinical setting rather than the current experiments mimicking the adjuvant setting. We conducted two experiments using doxorubicin and paclitaxel in the MDA-MB-231 model with inconsistent effects on lung fibrosis levels, suggesting that this will be a more complex pathway (data not shown). This is another area in which autopsy data could be insightful.

## MATERIALS AND METHODS

### Cell lines

Murine mammary carcinoma 4T1 luciferase-labeled (4T1-Luc2) cells (PerkinElmer, MA) were cultured in Dulbecco's modified Eagle medium (DMEM, Invitrogen, Frederick, MD) supplemented with 10% fetal bovine serum (FBS). A subline of human MDA-MB-231 cells, designated MDA-MB-231T was generously provided by Dr. Zach Howard (Laboratory of Immunoregulation, National Cancer Institute, Bethesda, MD) and was maintained in DMEM supplemented with 10% FBS. All ovarian cancer cell lines were provided by Dr. Annunziata (Women's Malignancies Branch, National Cancer Institute, Bethesda, MD) and cultured in RPMI-1640 media (ThermoFisher Scientific) supplemented with 10% FBS.

### Reagents

Lysophosphatidic acid (LPA) was purchased from Sigma-Aldrich (St. Louis, MO). Prior to use, LPA was dissolved in PBS containing 1% fatty acid-free bovine serum albumin.

LPA1 antagonists. SAR100842 was provided by Sanofi (Paris, France) and EPGN9878 was provided by Epigen Biosciences, Inc. (San Diego, CA). Powdered stock for each was stored in the dark at 4° C. For all *in vitro* experiments, SAR100842 and EPGN9878 were dissolved into 100% DMSO to 10 mM stock and further diluted in serum free media to indicated concentrations for downstream assays. For *in vivo* assays, SAR100842 was obtained as a 25% nanocrystal dispersion (NCD) at 50 g/50 mL directly from Sanofi. For dosing in mice, the NCD was further diluted in a vehicle of 0.6% methylcellulose-0.5% Tween-80 to 3 mg/mL. SAR100842 has a half-life of 4.9 h and a C_max_ of 5600 ng/mL after a 30 mg/kg oral dosing in mice. Mice received 30 mg/kg by oral gavage twice daily for the duration of the experiment. EPGN9878 powdered stock was dissolved to 4 mg/mL in 95:5 v/v 30% captisol (SBECD) solution and dimethylacetamide (DMA). Mice received 40 mg/kg by oral gavage once daily for the duration of the experiment. The diluent for each compound was used as its vehicle control.

IPF Drugs. Nintedanib was obtained from MedKoo Biosciences (Chapel Hill, NC), pirfenidone was obtained from MedChem Express (Monmouth Junction, NJ). Nintedanib was dissolved in 100% DMSO to 10 mM stock and pirfenidone was dissolved in 100% DMSO to 50 mM stock, and both were diluted in serum free media for all *in vitro* assays. For *in vivo* assays nintedanib was suspended in 0.5% hydroxyethyl cellulose and dosed at 50 mg/kg once daily by oral gavage, and pirfenidone was suspended in 0.5% carboxymethyl cellulose and dosed at 250 mg/kg twice daily by oral gavage. For each, the vehicle was the diluent.

### Western blots

Protein was extracted from all cell lines using standard RIPA buffer methods. Protein concentrations were estimated with BCA Protein Assay Kit (Thermo Scientific, Rockford, IL). SDS-Page was performed using the BioRad TGX system (Hercules, CA). Blots were blocked and incubated in primary antibody to LPA1 (Abcam) overnight, before incubation in a secondary HRP (Santa Cruz) followed by development with the Immobilon Western Chemiluminescent HRP Substrate system (EMD Millipore, Billerica, MA).

### *In vitro* functional assays

Cellular proliferation was measured by alamarBlue cell viability assay (ThermoFisher Scientific). Cells were trypsinized for approximately 3–5 minutes, Dulbecco's modified eagle medium (DMEM) containing 10% fetal bovine serum (FBS; Invitrogen, Carlsbad, CA) was added to neutralize the trypsin, cells were collected and counted. The cells were then plated at a density of 2,000 cells per well in a 96-well plate and allowed to attach overnight. AlmarBlue was added to cells at a 1:10 dilution in culture media and incubated at 37° C for 5 hours, at which time the fluorescent emission and excitation was measured on a SprectraMax M2 plate reader (Molecular Devices, Sunnyvale, CA) at 560 nm and 590 nm respectively. Cells were then treated with vehicle and increasing concentrations of drug and fluorescent readings taken at 24, 48 and 72 hours.

Cell migration assays were performed in Boyden chambers (Neuro Probe, Gaithersburg, MD) as previously described [68]. Briefly, cells were serum starved and incubated in vehicle or varying concentrations of drug for 24 hours. Lower wells contained DMEM with or without attractants (1–5% FBS or 5–10 μM LPA). 4T1-Luc2 or MDA-MB-231T cells were added to the upper wells at a concentration of 2 × 10^5^ cells/mL in serum-free DMEM containing vehicle or drug and incubated for 4 hours in a humidified chamber at 37° C in 5% CO_2_. The top chamber was removed and the cells that had migrated to the bottom of the membrane were stained using Diff Quick Staining kit (Invitrogen). Using an inverted brightfield microscope with a 10× objective, the number of cells that had migrated through the membrane was counted in three fields in the center of each filter. Each condition for all cell lines was assayed in triplicate and each experiment was independently performed three times.

For the wound healing assay, cells were plated at a concentration of 75,000 cells per well to 24-well plates in normal growth medium. Each cell line was plated in triplicate. Cells were grown for 24 hours to 100% confluence. Each well was scratched by a 1 mL tip in a vertical and horizontal direction to create a cross in the center of each well. After scratching, cells were washed with medium to remove the detached cells and replaced with fresh growth medium containing 1% FBS and increasing concentrations of LPA1 antagonist. Fresh media containing antagonist was replaced daily for the duration of the experiment. The time of initial scratch was stipulated as time 0. Pictures of scratches were taken by using phase contrast and 10× magnification at times: 0 h, 24 h, 48 h, and 72 h. Analysis was performed by loading images into ImageJ. Closed wound area was calculated as a percentage of area of wound at time X over area of wound at time 0 for each time point.

### *In vivo* studies

Frederick National Laboratory is accredited by AAALAC International and follows the Public Health Service Policy for the Care and Use of Laboratory Animals. Animal care was provided in accordance with the procedures outlined in the “Guide for Care and Use of Laboratory Animals” (National Research Council; 2011; National Academies Press; Washington, D.C.).

Experiments were performed under an approved National Cancer Institute Animal Use Agreement. Female six-week-old Balb/c or Balb/c nu/nu mice were obtained from Charles River Laboratories (National Cancer Institute-Frederick Animal Production Area, Frederick, MD). In all studies, mice were weighed weekly and monitored for signs of ill health and labored breathing daily. The mice were sacrificed by being placed in a carbon dioxide chamber if pathologic conditions unrelated to the study (eg, breathing difficulties) developed or if they lost more than 20% of their starting body weight.

### Spontaneous metastasis mouse model

For the spontaneous metastasis experiments, 5 × 10^5^ 4T1-Luc2 cells were injected into the mammary fat pads of female Balb/c mice (*n* = 15–20 mice per group) [18]. Randomization protocol and treatment schedules were the same for all antagonists tested. On day two post cell implantation, mice were randomized to three groups. Group one began vehicle on day 2, group two began treatment on day two and group three began treatment on day 11 (one day post primary tumor resection). The primary tumors were measured beginning on day four and then daily until resection, which occurred on day 10. After 8 weeks all lungs, livers, and any other organ suspected of harboring a metastasis were collected for histologic analysis. Drug treatment starting on day 2 post-injection of cells was for efficacy experiments and on day 11 post-injection of cells for adjuvant experiments.

### Experimental pulmonary metastasis mouse model

The experimental metastasis study was conducted using Athymic NCR nu/nu mice injected intravenously into the lateral tail vein with 7.5 × 10^5^ MDA-MB-231T human breast cancer cells [18]. On the day following injection, mice were randomized into two groups containing 15 mice each receiving either vehicle or drug. The study was terminated at day 70 and the lungs and liver were harvested from each animal. For each group, ten animals had the entire lungs inflated and fixed in Bouin's solution. Surface metastatic lesions were counted on all lungs using a magnifying glass, blinded to the treatment group. Any nodule larger than 5 mm in diameter was counted separately as a large metastasis.

For the remaining five animals from each of the groups, the left lung lobe and the left liver lobe were fixed-frozen using a 4% PFA sucrose gradient. The remaining lung and liver tissue was fixed in 10% NBF and switched to 70% EtOH 24 hours later for paraffin embedding. Tissues were also used for immunofluorescence or immunohistochemical staining for expression of different proteins.

### Ovarian model

To study SAR100842 in the ovarian cancer lines, 6–8 week old athymic nu/nu mice received either 1.0 × 10^6^ SKVO3 cells or 3.5 × 10^6^ OVCAR5 cells (*n* = 33 mice per line) in an intraperitoneal injection. On day two post cell injection, mice were randomized to three groups. Group one began vehicle on day 2, group two began 30 mg/kg SAR100842 twice daily on day two for the duration of the experiment, and group three began 30 mg/kg SAR100842 twice daily on day 10 for the duration of the experiment. On day 70 post cell injection all mice were euthanized and necropsied. Liver, abdominal lymph nodes or masses, omentum, peritoneum and any other organ suspected of harboring tumor were collected, fixed in 10% NBF and prepared for histological analysis. If present, ascites or peritoneal washings (1 mL) were collected and frozen.

### Histology

All paraffin-embedded tissues from were sectioned on a microtome. Six micron sections were taken at two hundred micron intervals through the entirety of the tissue. Sections were subsequently stained with hematoxylin and eosin. Metastatic lesions were counted under a microscope in each section, blinded for treatment group. For the ovarian models, disease was assessed as surface (tumor attached to the tissue) or invasive (tumor invading into the tissue), and number of animals per group recorded.

Lung sections stained with Masson's trichrome (Sigma Aldrich) were quantitatively assessed for severity of fibrosis using the Ashcroft score [31]. Briefly, 4–5 random fields of view were scored from 0 – 8 according to Ashcroft from sections taken from at least 5 mice per treatment or control group.

### Immunofluorescence

Formalin-fixed, paraffin embedded sections of lungs were sectioned (6 micron thickness) on a microtome. A minimum of one section from each of five mice per treatment group was stained. Briefly, sections were deparaffinized in xylenes, rehydrated in alcohols, and underwent antigen retrieval (Dako). Sections were then blocked for 1 hour at room temperature in PBS containing 5% goat serum (Dako Cytomation, Carpinteria, CA). Antibodies against αSMA (Abcam) and collagen 1 (Col1, Millipore) were incubated overnight at 4° C and then sections were washed three times in PBS. Secondary polyclonal Alexa Fluor antibodies to rabbit IgG diluted at 1:500 were incubated at room temperature for 1 hour before the sections were imaged using a fluorescent microscope (Zeiss, Oberkochen, Germany). 4’,6-diamidino-2-phenylindole was used to stain the cell nuclei.

### Image analysis

To quantitate αSMA positive staining, five random fields of a single lung tissue section per mouse were imaged at 10× objective. In each image, the area of metastasis was outlined and the percent of αSMA positive area was calculated as the area of positive fluorescent stain over total area outlined (ie: total metastasis area) using the image analysis function in Zen Imaging Software (Zeiss). The mean percent αSMA positive area per mouse was determined from the five fields.

### Statistical analysis

Statistical analysis was conducted using Prism 4 software (Graph Pad Software, Inc.) Significance between groups was determined using Student's *t*-test, ANOVA, or two-way ANOVA depending on the assay. *P*-values lower than 0.05 were considered significant.

## SUPPLEMENTARY MATERIALS FIGURES AND TABLES



## Abbreviations

DMA: dimethylacetamide
DMEM: Dulbecco's Modified Eagle Medium
DMSO: dimethylsufoxide
ECM: extracellular matrix
EMT: epithelial-mesenchymal transition
FBS: fetal bovine serum
H&E: hematoxylin and eosin
IP: intraperitoneal
IPF: idiopathic pulmonary fibrosis
IV: intravenous
LPA: lysosphosphatidic acid
LPAR: lysophosphatidic acid receptor
MFP: mammary fat pad
NCD: nanocrystal dispersion
PDGF: platelet-derived growth factor
SMA: smooth muscle actin
TGF: transforming growth factor
