# Supplementary material for: Limited fibrosis accompanies triple-negative breast cancer metastasis in multiple model systems and is not a preventive target
Source: Oncotarget. 2018 May 4;9(34):23462–81. doi: 10.18632/oncotarget.25231 (PMC5955109; doi:10.18632/oncotarget.25231)
Supplement: Supplementary file 1 [file oncotarget-09-23462-s001.pdf]

Limited fibrosis accompanies triple-negative breast cancer metastasis in multiple model systems and is not a preventive target

SUPPLEMENTARY MATERIALS

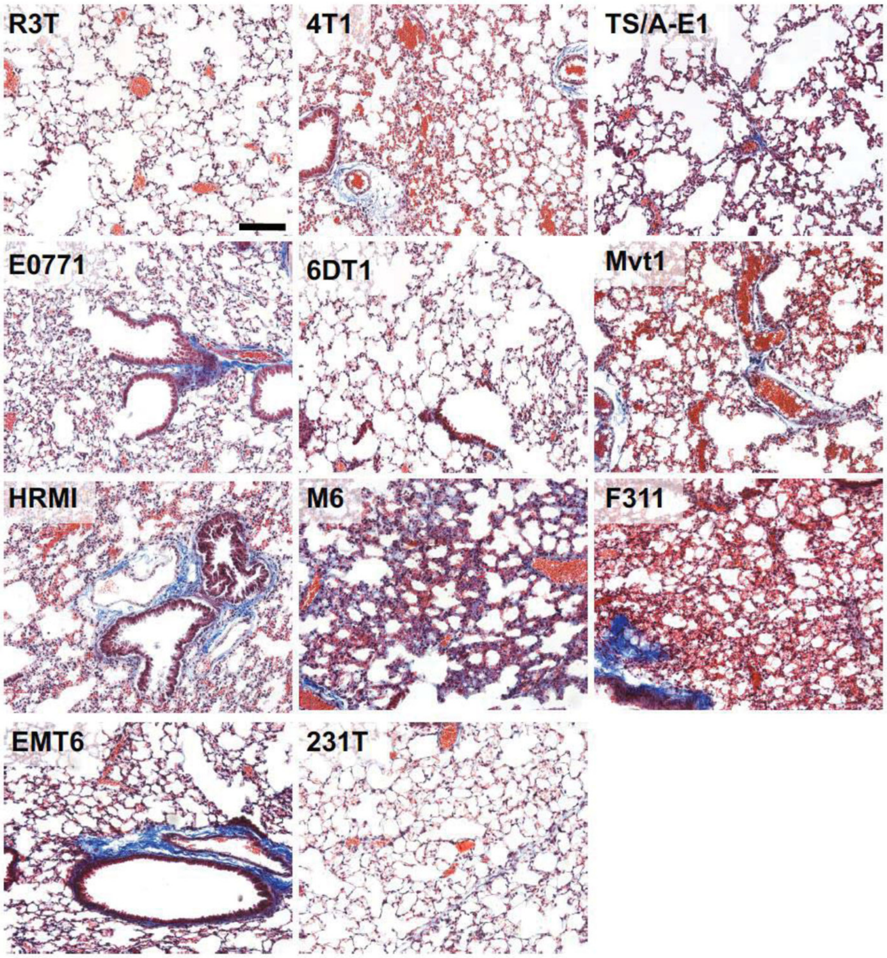

Supplementary Figure 1: Trichrome staining of normal or uninvolved lung. Formalin fixed, paraffin embedded (FFPE) lung tissue sections from metastasis bearing mice stained for Masson's trichrome. Unaffected lung adjacent to areas of metastatic lesion were analyzed for fibrosis using the normal Ashcroft scoring method to assess a lung score (Table 1). Representative images were taken of unaffected lung area adjacent to the metastatic lesions shown in Figure 1 at 20× objective, scale bar = 100 µm.

Supplementary Table 1: Ashcroft scoring system based on Masson's trichrome staining[a]

	Ashcroft Score[b]	Modified Ashcroft Score[c]
Score:	Description of Field	
0	Normal Lung	No evidence of fibrosis, normal lung
1	Minimal fibrous thickening of alveolar or bronchiolar walls	Minimal evidence of fibrosis surrounding and/or in metastatic lesions
2		
3	Moderate thickening of walls without obvious damage to lung architecture	Moderate evidence of fibrosis surrounding and/or in metastatic lesions
4		
5	Increased fibrosis with definite damage to lung structure and formation of fibrous bands or small fibrous masses	Moderate to severe evidence of fibrosis surrounding and/or in metastatic lesions
6		
7	Sever distortion of structure and large fibrous areas; "honeycomb lung" is placed in this category	Severe amount of fibrosis surrounding and/or in metastatic lesions
8	Total fibrous obliteration of the field	Total fibrous obliteration of the field

[a]One section with the largest representative lung area was stained with Masson's trichrome. Randomly, 4–5 fields of view were scanned and assessed a score from 0–8.

[b]For lungs with no metastases or to assess unaffected lung areas, normal Ashcroft score was used.

[c]For areas with metastatic lesions, a modified Ashcroft score was assigned.
